# COVID-19 Communication Ecology: Visualizing Communication Resource Connections During a Public Health Emergency Using Network Analysis

**DOI:** 10.1177/0002764221992811

**Published:** 2021-02-05

**Authors:** J. Brian Houston, Esther Thorson, Eunjin (Anna) Kim, Murali K. Mantrala

**Affiliations:** 1 University of Missouri, Columbia, MO, USA; 2 Michigan State University, East Lansing MI, USA; 3 University of Southern California, Los Angeles, CA, USA; 4 University of Kansas, Lawrence, KS, USA

**Keywords:** communication ecology network (CEN) model, network analysis, COVID-19, communication resources

## Abstract

The COVID-19 outbreak began in December 2019 and soon became a global pandemic, resulting in major public health consequences for countries across the world. As the COVID-19 outbreak evolved, individuals were challenged to understand the risk of COVID-19 and to identify ways to stay safe. This understanding was accomplished through COVID-19 communication ecologies that consist of interpersonal, organizational, and mediated communication resources. In the current study, we examine the U.S. COVID-19 communication ecology in September 2021. We introduce the communication ecology network (CEN) model, which posits that similar useful communication resources will cluster in a communication ecology, and we use network analysis for visualization of the ecology. Our results indicate a robust COVID-19 communication ecology. The most important communication resources in the ecology were partisan and organizational communication resources. We identify and discuss five clusters within the COVID-19 communication ecology and examine how use of each of these clusters is associated with belief in COVID-19 misinformation. Our use of network analysis illustrates benefits of this analytical approach to studying communication ecologies.

A new coronavirus, SARS-CoV-2, emerged in December 2019 to quickly become a global public health threat (Fauci et al., 2020). As of February 2021, the respiratory disease caused by this novel virus (COVID-19) has resulted in multiple waves of outbreaks in the U.S. resulting in over 440,000 deaths and more than 25 million cases (https://covid19.who.int/region/amro/country/us). In addition to death and physical illness, COVID-19 has exerted a significant public mental health impact, resulting in increased individual stress, anxiety, depression, and grief reactions (Eisma & Tamminga, 2020; First et al., 2020).

As COVID-19 emerged and began to impact society, individuals were challenged to quickly understand the threat of this new virus and determine how to stay safe. In terms of understanding the COVID-19 threat, this meant that people needed to learn how severe the COVID-19 disease was and how susceptible they (and their families) were to the illness. Additionally, individuals also then needed to determine how best to protect themselves (and loved ones) from the disease. Ascertaining COVID-19 risk is potentially complicated because some individuals infected with COVID-19 may not experience any symptoms, while others may experience severe symptoms that can result in hospitalization and death (Beck & Aksentijevich, 2020). As a result of this range of possible disease impacts, personal COVID-19 risk severity and susceptibility may not always be clear. Ascertaining the best protective behaviors to utilize during the COVID-19 pandemic can also be difficult, as public health recommendations related to COVID-19 often changed in real time as scientific understanding of the disease evolved. For example, early in the pandemic many public health agencies did not recommend the wearing of masks to protect against COVID-19, though that recommendation later changed (Yan, 2020). Recommended quarantine times also changed (Rodriguez, 2020).

During a public health emergency like COVID-19, individuals may seek to understand an emerging disease through an evolving communication ecology that includes interpersonal, organizational, and mediated communication resources. The current study examines the use of a variety of COVID-19 communication resources by U.S. adults to gather information about the novel and evolving COVID-19 pandemic. In studying this case, we introduce the communication ecology network (CEN) model, which posits that utilization of related communication resources will cluster together within an overall CEN. To examine this model, we conduct a preliminary demonstration of network analysis as a novel approach to analyzing a communication ecology. Overall, this article contributes to theoretical understanding of communication ecologies related to a global public health emergency and demonstrates a new method of analyzing communication ecologies. To begin, we review the concept of communication ecology.

## Communication Ecologies

Communication ecologies are “the networks of communication connections that groups or individuals depend upon in order to achieve a goal” (Broad et al., 2013, p. 328). Communication ecologies can include any communication resources that an individual or group might use to access or share information, and include both mediated and nonmediated resources (Hearn & Foth, 2007). Traditional media (broadcast, print), social media, technological devices and systems, organizational sources, and interpersonal sources are potentially components of a communication ecology (Houston et al., 2015; Wilkin, 2013). Communication ecologies are specific to communication goals, so for example, the communication ecology assembled by an individual or group to engage health issues (Wilkin, 2013) may or may not differ from the communication ecology an individual or group constructs to cope with disasters (Spialek & Houston, 2017) or take political action (Mercea et al., 2016). Individual dependence on a communication resource is related to the “perceived utility” of the resource to facilitate goal attainment (Ball-Rokeach, 1985, p. 495). In addition, beyond serving individual or group goals, communication ecologies can provide capacity for collective (community) planning, action, reflection, and problem solving (Houston et al., 2015; Mercea et al., 2016).

Given the goal orientation of communication ecologies, we propose the CEN model, which posits that utilization of related resources will cluster together within a CEN. Communication ecologies have a networked structure, meaning that use of one communication resource within a communication ecology may lead to the use of another resource, and vice versa. In terms of achieving goals, a connected set of communication resources may be more useful for individuals than relying on a single resource. This is because use of multiple communication resources, compared with use of a single or a few communication resources, may provide an individual with additional information that can be used to inform behavior. Using more communication resources may also provide information triangulation (Greyson, 2018), which can provide an individual more confidence that the information received is accurate and trustworthy.

From a CEN perspective, when communication resources cluster, it means that individuals are more likely to use these different communication resources together. That is, the use of one communication resource in the cluster may be associated with (or lead to, or result from) the use of another. For example, an individual using Facebook to find out about an issue may often be exposed to and click on links to online newspaper stories about that issue. In such an example, use of Facebook and online newspapers may cluster. Similarly, when communication resources are less clustered with other resources, then they are less likely to be used with those other resources. A variety of communication resources may exist within a communication ecology, and these resources may be connected to other ecological resources in different ways. This is an aspect of communication ecologies that has not been examined in previous research.

### Communication Ecology Levels

A communication ecology perspective often considers different levels of communication resources, generally ranging from the macro to micro levels (Kang, 2016). Macro-level resources can include international or national mediated sources, such as television networks and newspapers (e.g., *The New York Times, BBC, Al Jazeera*) that provide significant coverage of events occurring across the nation or world. Conversely, a micro-level communication resource can include hyperlocal websites and interpersonal communication with friends and family. The different levels of resources in a communication ecology can be useful, but in terms of understanding one’s local community (town, neighborhood), more micro-level communication resources may often be most relevant (Ball-Rokeach et al., 2001). This is because macro information sources do not typically address local conditions, issues, or occurrences. At the same time, macro-level communication resources may usually prove most helpful for individuals seeking information about national events or issues (Kim et al., 2002).

### Communication Ecologies During Public Health Crisis

In terms of helping explain how and why individuals depend on different communication resources, media dependency theory posits that during a social crisis, individual use of media resources increases due to a heightened need for information to make sense of an unstable social situation (Ball-Rokeach, 1985; Lowrey, 2004). A public health crisis is one example of a collective event that may increase dependence on and use of communication resources. The heightened threat and uncertainty resulting from an emerging disease is likely to motivate individuals to construct communication ecologies to gain information about the illness. The case of COVID-19 is a novel case in which to explore a communication ecology, because while it constitutes a local, community (micro) crisis (in that COVID-19 affects the local community), it also receives significant national and international (macro) media coverage as a global pandemic. Thus, all levels of communication resources are potentially useful. For this reason, the current study considers the different *levels* of communication ecologies during a global pandemic. Per media dependency theory, we expect individuals to be highly engaged with communication resources at multiple levels related to COVID-19 in order to make sense of the crisis. We utilize network analysis in this study, providing a preliminary demonstration of a novel approach to studying communication ecologies.

## Network Analysis

Communication ecologies are conceptualized as networks of communication connections (Broad et al., 2013), and network analysis is a useful analytical approach for investigating the pattern of these connections. Networks are models of systems, wherein individual entities (nodes) are connected (by edges) that can be graphed to visualize a system (Newman, 2010). A network approach to analyzing communication behaviors can capture and represent the interactive or reciprocal nature of communicative actions. For example, the use of Facebook to gain information about an issue or event may facilitate the use of an online newspaper that a Facebook post links to, or Facebook use may foster interpersonal discussion with others (online or in-person) about an issue or event addressed in a post. These common patterns of communication resource behavior may become visible with network analysis.

In helping understand a specific construct, network analysis illuminates the architecture of a *system* (Schmittmann et al., 2013). Moreover, in network analysis the variables analyzed “do not measure the construct, but are a part of it” (Schmittmann et al., 2013, p. 47). In this way, network analysis differs from other reflective analytical approaches such as latent variable analysis, where variables are thought to reflect an underlying construct, and from formative analytical approaches such as factor analysis, where variables are considered to determine the construct (Schmittmann et al., 2013). As such, network analysis is an appropriate, practical, and novel approach to studying interconnected systems of communication resources.

Additionally, a network approach to assessing communication ecologies allows many different communication resources to be considered and potentially retained in the final network model. At the same time, network analysis allows for the detection of communication resource behaviors that are particularly important (or central to the network) in terms of acting toward or achieving the goal that is related to the communication ecology.

In psychology and psychiatry, network models have been used to examine the interrelationships of attitudes (Dalege et al., 2017), symptoms, and behaviors (DuBois et al., 2017; McBride et al., 2020; van Zyl, 2018). With regard to communicative behaviors, Yun et al. (2018) used network analysis to explore empathetic communication behaviors (with patients) among medical students. This analysis detected several behavioral and cognitive hubs for fostering empathetic communication among medical students, with the most important being stress management strategies (to address feelings of agitation that impeded empathetic communication among participants). These results indicate the utility of a network approach to conceptualizing complex and multifaceted human communication behaviors. To our knowledge however, network analysis has not yet been applied to the study of a communication ecology, even as scholars have called for new analytical methods to assess communication ecologies (Shah et al., 2017).

While social network analysis is a popular type of network analysis in communication research, social network analysis specifically addresses the structures of *social* relationships (Shumate & Palazzolo, 2010). So, for example, a study employing social network analysis might investigate how social media users (or accounts) are connected through interaction in an online social network (e.g., Bennett et al., 2014). Thus, social network analysis models connections between individuals or groups. In the CEN analysis presented in the current study, we instead illustrate how the use of different *communication resources* are connected using survey data of individuals reporting that use. To guide our network analysis, we pose the following research question:

**Research Question 1:** What is the network structure of the COVID-19 communication ecology?

Following network analysis, we also explore how COVID-19 beliefs are associated with use of the COVID-19 communication ecology. This provides initial insights into possible effects that result from individuals using the COVID-19 communication ecology. Specifically, we examine belief in COVID-19 misinformation. The COVID-19 global pandemic has been described as an *infodemic* that includes a large amount of misinformation about the disease, and belief in this misinformation among individuals has hampered the public health response to the outbreak (Garrett, 2020; Zarocostas, 2020). Ideally, an effective communication ecology during a pandemic would provide factual information to individuals, which in turn informs accurate beliefs about the disease. However, it is also possible that a communication ecology includes misinformation that informs inaccurate beliefs. We explore the association between use of the COVID-19 communication ecology and accurate COVID-19 beliefs in the current study:

**Research Question 1:** How is COVID-19 communication ecology use related to belief in COVID-19 misinformation*?*

## Method

To answer our research questions, we conducted a survey of *N* = 644 U.S. adults (aged 18 years or older) in the states of Michigan and Texas. We focused on two U.S. states that had earlier (Michigan) and later (Texas) experiences with a COVID outbreak in 2020. Data were collected September 7-18, 2020. During the time of the survey, Texas was approaching the peak of its second wave of COVID-19, while rates of COVID-19 remained much lower in Michigan following an earlier, severe spike of disease outbreak. Participants were recruited via a Qualtrics Panel aggregator system. Potential respondents were sent an email invitation with a secure URL to review the study’s purpose and access the survey. An electronic informed consent indicated that participation was voluntary and responses would be anonymous. After consenting to the study, participants were directed to the online survey. Participants were compensated for their time with incentives through the Qualtrics incentive program, which includes prize drawings and cash incentives.

### Participants

Participants reported their gender as 50.6% female (*n* = 326), 49.2% (*n* = 317) male, and 0.2% (*n* = 1) nonbinary. Overall, 76.1% of participants reported their race as White (*n* = 490), 8.1% as Black (*n* = 52), 7.6% as Hispanic or Latinx (*n* = 49), 0.6% as American Indian or Alaska Native (*n* = 4), and 1.6% as another race or multiracial (*n* = 10). Regarding age of participants, 19.6% were 18 to 29 years (*n* = 126), 26.2% were 30 to 44 years (*n* = 169), 24.2% were 45 to 59 years (*n* = 156), and 30.0% were 60 years and older (*n* = 193). Highest education level of participants was reported by 3.0% as some high school or less (*n* = 19), 37.4% as high school graduate (*n* = 241), 25.8% as some college (*n* = 166), 21.4% as a college graduate (*n* = 138), and 12.4% as a graduate degree (*n* = 80).

### Measures

#### COVID-19 Communication Ecology

To assess the COVID-19 communication ecology, we asked participants how often in the last week they had used 27 different sources to find out about the COVID-19 pandemic or virus on any device (e.g., television, computer, iPad, phone). Response options ranged from 1 (*never*) to 5 (*very often*). Each communication resource item included examples as appropriate. Communication resources assessed included news organizations (e.g., national television news, FOX cable news programs, local newspapers), social media platforms (e.g., FaceBook, YouTube, Reddit), government agencies (e.g., public health websites such as the Centers for Disease Control and Prevention), organizations (e.g., local organizations helping the community, faith-based organizations), and friends and family. See Table 1 for full list of all 27 survey items and descriptive results.

**Table 1.** COVID-19 Communication Ecology Network Details.

Node abbreviation	Node name	Node survey item (variable)	Node degree	Frequency of use	
				*M*	*SD*
NTV	National TV news	National news on *CBS, ABC*, or *NBC*	3	3.07	1.43
LTV	Local television news	Local television news	3	3.24	1.40
CNN	CNN programs	*CNN* cable news programs (e.g., Wolf Blitzer, Don Lemon)	4	2.21	1.43
LTN	Local TV programs	Local television news programs	3	3.00	1.43
FOX	FOX programs	FOX cable news programs (e.g., Rachel Maddow, Chris Matthews)	5	2.24	1.43
MSN	MSNBC programs	MSNBC cable news programs (e.g., Rachel Maddow, Chris Matthews)	7	1.94	1.26
CON	Conservative news	Conservative news websites (e.g., *One America News Network* (ONA), *Breitbart, The Daily Caller*)	6	1.77	1.16
LIB	Liberal news	Liberal news websites (e.g., *Huffington Post, Politico, Daily Kos*)	8	1.79	1.17
RAD	Conservative talk radio	Conservative talk radio, podcasts, streaming service (e.g., Rush Limbaugh)	5	1.75	1.19
NNW	National newspapers	National newspapers (e.g., *The New York Times; The Washington Post*; online or print)	10	1.97	1.29
LNW	Local newspapers	Local newspapers (online or print)	4	2.46	1.39
FB	Facebook	Facebook	3	2.47	1.46
TWT	Twitter	Twitter	7	1.89	1.38
YT	YouTube	YouTube	7	2.13	1.42
RED	Reddit	Reddit	8	1.59	1.16
GOV	Government websites	Official government websites (e.g., city government websites, state government websites)	6	2.13	1.24
CDC	Public health websites	Public health websites (e.g., state health department, CDC, World Health Organization)	6	2.13	1.22
CEL	Celebrities	Celebrities that you admire and like	10	1.63	1.12
FAM	Friends and family	Friends and family	5	2.93	1.23
Q	QAnon	QAnon and other groups focused on investigating the deep state	10	1.54	1.06
JOE	Joe Rogan	The Joe Rogan Experience podcast	9	1.50	1.02
BLM	Black Lives Matter	Protest groups like Black Lives Matter	11	1.72	1.19
DOC	Healthcare provider	Your local doctor or healthcare provider	7	2.52	1.33
IND	Independent news sources	Independent news sources in your state like *The Texas Tribune* or *The Bridge in Michigan*	8	1.61	1.09
ORG	Local organizations	Local organizations helping the community like YMCA, Boys and Girls Clubs, Food Bank, Salvation Army, etc.	11	1.57	1.03
FTH	Faith-based organizations	Your local church, synagogue, mosque, or temple	7	1.72	1.14
SCH	Schools	Your local school system or university	10	1.81	1.21

*Note*. Table includes the node abbreviations used in the network analysis, a brief node name for each item used in the manuscript text, the full survey item for each variable used in the survey, the degree for each node (i.e., the number of other nodes each node is connected to in the network analysis results), and the mean and standard deviation for each survey item.

#### COVID-19 Misinformation

Belief in COVID-19 misinformation was assessed by asking participants to indicate whether six misinformation statements about COVID-19 were true or false. These items were developed for this study based on COVID-19 misinformation circulating at the time of the survey. Example statements included, “the ‘fake pandemic’ is part of a global effort to force everyone to be vaccinated,” “wearing a mask traps the carbon dioxide you breathe out and can make you ill,” and “the symptoms that most people blame on COVID-19 appear to be connected to 5G network radiation.” Response options ranged from 1 (*definitely false*) to 5 (*definitely true*). For analysis, the mean was calculated for all items and a higher score indicated stronger misinformation beliefs (*M* = 2.56, *SD* = 0.96, α = .82).

### Data Analysis

#### Network Estimation and Visualization

To estimate the COVID-19 CEN (the network of communication resources related to COVID-19), we used a Pairwise Markov Random Field model (van Borkulo et al., 2014). A Pairwise Markov Random Field model approach estimates a network wherein the nodes (communication ecology survey items) are connected by edges with no arrowheads (undirected edges; Epskamp et al., 2018). When an edge connects nodes, this indicates a conditional dependence. When nodes are not connected, then they are independent of each other. To develop a parsimonious and more interpretable CEN, we converted our communication ecology question items into binary data for network analysis (Yun et al., 2018). To do this, we recoded all communication ecology responses of *never* and *rarely* to 0 (*absence of that behavior*), and we recoded all responses of *sometimes, often*, and *regularly* to 1 (*presence of that behavior*).

We estimated the binary CEN using the eLasso procedure (van Borkulo et al., 2014) in the R package *IsingFit* (van Borkulo & Epskamp, 2015). This procedure regresses every variable on all other variables in the network, and then each regression function is regularized to address any problems with multicollinearity (Friedman et al., 2008). The independent variables in each regression represent the nodes that the dependent variable is connected to by edges, and the edges are weighted based on the regression parameters (Dalege et al., 2017). This provides an overview of how the data are associated with each other in the data set (Epskamp et al., 2012). For binary data network analysis, smaller sized networks (consisting of 10-30 nodes) require a sample size of 500 (van Borkulo et a., 2014), and thus our sample is sufficient for this analysis.

For network estimation, the *IsingFit* (van Borkulo & Epskamp, 2015) package produces a weight adjacency matrix that contains the edge weight for each set of nodes in the network (Dalege et al., 2017). We then plotted the estimated network using the R package *qgraph* (Epskamp et al., 2016). The network is plotted with the Fruchterman-Reingold algorithm (Fruchterman & Reingold, 1991), which positions nodes that are strongly connected to each other more closely together. Thicker lines in the plot represent stronger connections between nodes, while thinner lines indicate weaker connections. We also included community (or cluster) detection in the plotting using the walktrap algorithm (Pons & Latapy, 2005) in the R package *igraph* (Amestoy et al., 2015). Community detection plotting represents clusters of strongly connected nodes within the network using different colors (e.g., if two communities are identified then black nodes indicate one cluster and white nodes indicate another cluster).

#### Centrality Estimation

Following the network estimation and visualization, we also examined centrality estimates. Centrality estimates indicate the importance of nodes within the network. We used the R package *qgraph* (Epskamp et al., 2016) to calculate and plot three centrality measures: strength, betweenness, and closeness. Strength is the sum of the absolute value of all of the edges connected to a network, which indicates the influence a specific node has on that network (Dalege et al., 2017; Dubois et al., 2017). Betweenness indicates how often a node sits on the shortest path between two other nodes in a network. Closeness is the average distance from one node to all of the other nodes in a network. Thus, closeness is an assessment of how often a node might be activated in a network as part of overall activity, and betweenness is a measure of how often a node might disrupt or facilitate activity flowing through the network (Dalege et al., 2017; McNally, 2016). The centrality indices were plotted with standardized z-scores, for which a higher value indicates more centrality.

#### Network Stability

In addition to assessing the importance of nodes in the network, we also examined the stability of the overall network. Centrality stability is calculated by examining the stability of centrality indices using subsets of the data to estimate the network. The R package *bootnet* (Epskamp et al., 2018) was used to calculate the *CS*-coefficient, which quantifies the highest proportion of cases that can be dropped to maintain a correlation of 0.7 with 95% certainty (Epskamp et al., 2018). The more cases that can be dropped and still be strongly correlated with the full network estimation indicates a more stable network (McBride et al., 2020). A preferred cutoff for the *CS-*coefficient is a value greater than 0.5 (Epskamp et al., 2018).

#### Network Effect

Finally, in order to explore the effect of COVID-19 communication ecology use, we used linear regression to examine the influence of sociodemographics and COVID-19 communication ecology on misinformation beliefs. We calculated a regression model that included participant age, gender, education level, political affiliation, and race/ethnicity in the first two blocks of the model. In the third block, we included the summed items for each communication ecology cluster identified in the network analysis.

## Results

**Figure 1.**
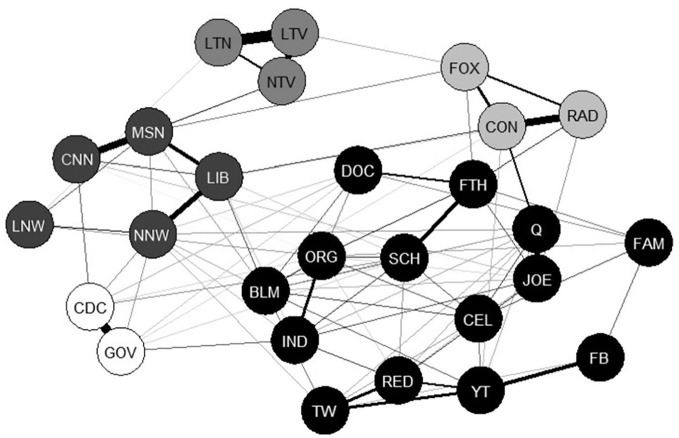
COVID communication ecology network. *Note*. Each node represents one questionnaire item assessing frequency of communication resource use related to the COVID-19 pandemic. See Table 1 for the node name and survey item that corresponds to each node label abbreviation. Thicker lines represent stronger connections between nodes compared with thinner lines. Node color indicates different communities within the network. Five clusters are shown with different node colors (white, black, dark gray, medium gray, light gray).

The network structure of the COVID-19 CEN is shown in Figure 1. This figure illustrates the associations between communication resource behaviors related to finding out about COVID-19 reported by participants from the U.S. states of Michigan and Texas. Connected nodes indicate individual communication resource behaviors that are associated with each other. We included 27 different communication resource in our original network estimation, and use of all of the resources were significantly connected to the network. Our results indicate that each communication resource behavior is connected to at least three other resources (nodes).

Following network estimation, we assessed the network for communities using the walktrap algorithm (Pons & Latapy, 2005) and identified five different clusters of nodes within the network (see Figure 1). A first cluster is indicated in Figure 1 with light gray nodes and includes conservative communication sources (*FOX News, Talk radio*, conservative websites). A second cluster is shown with medium gray nodes and includes TV news (local and national, and local TV news programs). A third cluster in dark gray includes liberal communication sources and newspapers (*MSNBC News*, liberal websites, *CNN* news, local and national newspapers). A fourth cluster is shown with white nodes and includes public health resources (government and public health websites). A fifth cluster is shown with black nodes and includes a diverse set of communication sources, including social media (e.g., Twitter, FaceBook), friends and family, local organizations, and partisan sources (e.g., Black Lives Matter, QAnon).

### Centrality Estimation

**Figure 2.**
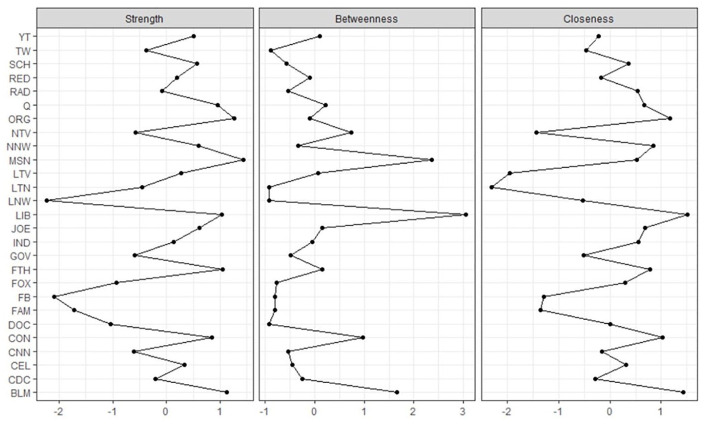
Centrality estimates for COVID communication ecology network. *Note*. See Table 1 for the node name and survey item that corresponds to each node label abbreviation. Centrality indices are plotted using standardized *z* scores.

Centrality estimates are plotted in Figure 2. Liberal websites were perhaps the important communication resource in the network in terms of all centrality measures (strength, betweenness, and closeness). *MSN* news and Black Lives Matters were also high on all measures.

### Network Connectivity and Stability

Network connectivity resulted in *L* = 1.93. A network is more connected when the value of *L* is lower. The network stability analysis indicates that betweenness, *CS*(cor = 0.7) = 0.75; closeness, *CS*(cor = 0.7) = 0.75; and strength, *CS*(cor = 0.7) = 0.60 are all above the preferred threshold of 0.5 (Epskamp et al., 2018). Thus, overall the network is well-connected and stable.

### Network Effect

In order to explore effects of using the COVID-19 communication ecology, we used linear regression to assess the relationship between participant sociodemographics and communication ecology use on belief in COVID-19 misinformation. We included sociodemographics in the first two steps of the model. In the final step, we calculated a mean score for all items in each COVID-19 communication ecology cluster shown in Figure 1 (television cluster, *M* = 3.10, *SD* = 1.28; liberal cluster, *M* = 2.07, *SD* = 1.03; conservative cluster, *M* = 1.92, *SD* = 1.09; public health cluster, *M* = 2.13, *SD* = 1.15; local and social cluster, *M* = 1.90, *SD* = 0.87). Results indicate that being more liberal (β = −.208, *p* < .001) and more educated (β = −.108, *p* = .010) was associated with less belief in more COVID-19 misinformation (see Table 2). With regard to the COVID-19 communication ecology, more use of the public health cluster (β = −.131, *p* = .002) and liberal cluster (β = −.243, *p* = <.001) was significantly associated with less belief in COVID-19 misinformation. Conversely, more use the conservative cluster (β = .413, *p* = <.001) and the local and social cluster (β = .213, *p* = <.001) was significantly associated with belief in more COVID-19 misinformation. Use of the television news cluster (β = −.072, *p* = .065) did not have a significant effect on belief in COVID-19 misinformation.

**Table 2.** Sociodemographic and Communication Ecology Predictors of Belief in COVID-19 Misinformation.

	Belief in COVID-19 misinformation	
	β	*p*
Step 1: Demographics		
Gender (female)	.013	.745
Age	−.040	.345
Education level	−.108	.010
Political affiliation (Democrat)	−.208	<.001
*R*^2^ = .16		
Step 2: Race/ethnicity		
Black	.049	.164
Asian	.009	.796
Hispanic	.080	.022
Native American	.000	.990
Other Race	−.004	.914
White (Reference)	—	—
Δ*R*^2^ = .01		
Step 3: Communication ecology use		
Television news cluster	−.072	.065
Public health cluster	−.131	.002
Liberal cluster	−.243	<.001
Conservative cluster	.413	<.001
Local and social cluster	.213	<.001
Δ*R*^2^ = .16		
Total *R*^2^ = .33		
*F*(14, 642) = 22.16, *p* < .001		

## Discussion

To understand how individuals used different communication resources to find out about a global public health emergency, we conducted an online survey of U.S. adults in the states of Michigan and Texas in September 2020. We used network analysis, a novel approach to studying communication ecologies, to estimate and plot a COVID-19 Communication Ecology (see Figure 1). We included interpersonal, organizational, and mediated communication resources in our assessment. These communication resources provided opportunities for individuals to obtain information about the COVID-19 pandemic.

Our results indicate that all of the communication resources we assessed constituted part of an overall communication ecology related to COVID-19. Use of every communication resource was related to at least three other communication resources. From a network perspective, the most important nodes in the COVID-19 communication ecology were partisan, organizational, and faith-based communication resources. For example, the strongest node in the network was *MSNBC news*, followed by local organizations, Black Lives Matter, faith-based organizations, liberal news websites, and QAnon. Similar patterns were observed for node betweenness and closeness, where liberal news websites, *MSNBC news*, Black Lives Matter, conservative news websites, and local organizations were important. Thus, partisan and local organizational (including faith-based organizations) communication resources had the most influence in the COVID-19 communication ecology and were the types of resources that were most likely to facilitate activity across the communication network.

In the United States, understanding of and behavior related to COVID-19 has been found to differ based on political affiliation, and this may explain the important of partisan communication resources in our results. For example, individuals who identified as political conservatives perceived COVID-19 to be less severe and reported less personal vulnerability to the disease compared with liberals (Calvillo et al., 2020). U.S. state public health responses to the pandemic have also differed based on partisanship. Democratic governors moved more quickly to make emergency declarations related to COVID-19 than did Republican governors (Fowler et al., 2020). Thus, if COVID-19 beliefs and behaviors are observed to organize according to political ideology, it makes sense that partisan communication resources would be important in COVID-19 communication ecologies. And we observe this importance in our results, as both conservative and liberal communication resources were found to exert strong influence on the network and facilitate communication activity across the network. Additionally, during a public health emergency, local organizations may be important and trusted sources of information and resources (Weinberger-Litman et al., 2020), which explains their importance in the COVID-19 communication ecology observed here.

### Communication Ecology Versus Frequency of Use

In terms of conceptualizing the importance of individual communication resources, our network approach produced different results than would likely have been identified through other statistical approaches. For example, descriptive statistical analysis (see Table 1) indicates that television news (local and national) were the most frequently used communication resources among our participants, followed by friends and family, healthcare providers, and Facebook. However, these resources were not identified in the network analysis as being strong. At the same time, most of the important communication resources in our network analysis were used less frequently by participants across our sample.

To investigate this pattern further in post hoc analysis, we calculated a Pearson correlation coefficient examining the relationship between how frequently individuals reported using communication resources (the mean score reported in Table 1) and the node degree (also reported in Table 1) for the communication resources (i.e., how many other communication resources each individual communication resource was connected to) resulting from our network analysis. We found a strong negative relationship between these two measures (*r* = −.785, *p* < .001). This indicates that the communication resources that were used most frequently tended to be connected to fewer other resources in the network, and that communication resources that were reported to be used much less frequently were generally connected to more resources in the model. In the case of COVID-19 then, some communication resources such as television news were used more often, but using these resources was not associated with using many other sources of information. This lack of connection may be structural, in that the act of watching television may be an activity that does not lend itself easily to using other communication resources. Or the lack of connection may be related to the limits of attention, in that when individuals spend more time with a single information source, they have less time to use other sources. Or viewers of television news might believe that they receive all or most of the information they need from this source (e.g., Shearer, 2020) and that they trust this news (Jurkowitz et al., 2020), and thus do not seek out additional information from other sources. Future research should examine the mechanisms of communication resource connection within a communication ecology to explore these and other possibilities.

### Communication Clusters Within the Network

Within this COVID-19 Communication Ecology, our network analysis identified five clusters (see Figure 1). Two of the clusters included more partisan sources, with a conservative cluster (shown in light gray in Figure 1) and a liberal cluster (shown in dark gray in Figure 1). The liberal cluster also included newspaper sources and *CNN*, and was thus more expansive than the conservative cluster. These two clusters were somewhat distant from each other in the ecology, though there were connections between *MSNBC news* and *FOX news* and between liberal news websites and conservative news websites. As discussed previously, partisan sources were important for the overall COVID-19 communication ecology, though these sources also largely clustered along ideological lines within the communication ecology.

Television news (local and national) constituted its own cluster (show in medium gray in Figure 1) that was weakly connected to the rest of the communication ecology. As noted earlier, television news was used frequently by participants, but use of television news was not strongly associated with using other communication resources. As discussed previously, given the frequency of television news use and the amount of trust is engenders (e.g., Jurkowitz, et.al., 2020; Shearer, 2020), many mean people may not supplement its use with other sources.

Another cluster included public health resources (shown in white in Figure 1). This was the smallest cluster in the network and included government and public health websites. There were multiple connections between the public health cluster and the rest of the network, though visually it is also evident that this cluster was distinctly separate from the rest of the ecology. From a public health perspective, this result is somewhat problematic. Ideally, official public health sources would be central to a communication ecology during a pandemic. However, this is not what we see in our results.

Finally, the last cluster included a variety of mediated, organizational, and interpersonal communication resources (shown in black in Figure 1). This cluster included some partisan mediated communication resources (e.g., QAnon, Black Lives Matter), local organizational resources (e.g., schools, faith-based organizations), interpersonal resources (e.g., friends and family), and social media (e.g., Twitter, Facebook). Visually, this cluster serves as the core of the network, with the other clusters orbiting. This cluster includes both micro- and macro-level resources. Micro-level resources include friends and family, local healthcare provider, and local faith-based organizations. Macro-level resources include *The Joe Rogan Experience* podcast (with a national and potentially international audience) and global social media platforms such as Facebook and Twitter.

The confluence of micro- and macro-resources in a single cluster illustrates the potential complexity of communication ecologies in the current media and social environment. These results also indicate scholars should better consider what is meant by communication levels in this environment. Facebook is a global social media platform, but a single individual user might largely use the platform to connect with friends and family and local organizations. So, should scholars consider a communication resource like Facebook to be macro or micro? More work is needed to explore this question and identify answers. Similarly, Black Lives Matter and QAnon are groups with national scopes, but they also operate locally in many cases. Again, are these macro or micro communication resources? Methodological and theoretical advances are need to better understand how these communication resources function in modern communication ecologies.

### COVID-19 Communication Ecology Effect

The COVID-19 pandemic has been called an infodemic of inaccurate and false information (Garrett, 2020; Zarocostas, 2020). Thus, we examined how use of different clusters in the COVID-19 communication ecology was associated with individual beliefs about COVID-19. We found that use of different portions of the ecology were related to both accurate and inaccurate COVID-19 beliefs. In terms of accurate COVID-19 beliefs, use of the public health cluster and liberal cluster were associated with less belief in COVID-19 misinformation. Although causality cannot be known using our cross-sectional data, this association suggests that individuals using these communication clusters were more likely to be receiving and believing accurate information.

Conversely, use of the conservative cluster and the local and social cluster was associated with more belief in COVID-19 misinformation. If inaccurate beliefs about COVID-19 lead to maladaptive health behavior, then these portions of the COVID-19 communication ecology are problematic from a public health perspective. The local and social cluster is perhaps particularly troubling, as it includes a variety of local resources (e.g., friends and family, schools, faith-based organizations, community organizations) that should be sources of useful and accurate information during a community public health crisis. Perhaps the social media sources that are also found in this cluster are the primary source of misinformation that then potentially circulates through the rest of this cluster. More research is needed to understand how this cluster functions during a public health crisis.

Last, the television news cluster was not significantly associated with accurate or inaccurate COVID-19 beliefs. Thus, we found that the communication resources that are most frequently used by individuals and that are most disconnected from the rest of the ecology are not contributing to accurate COVID-19 beliefs. From a public health perspective, this is a problematic aspect of the COVID-19 communication ecology.

### Understanding Communication Ecology Network Analysis and Future Research

The preliminary demonstration of network analysis to assess a communication ecology that is presented here warrants further discussion. Even though communication ecologies are conceptualized as networks of communication resources (Broad et al., 2013), a network approach has not been used as an analytical approach to understand ecologies prior to the current study. In the CEN analysis presented here, we illustrate how the use of different communication resources are connected using survey data of individuals reporting that use. We began this analysis with the CEN proposition that use of different communication resources by individuals will cluster within the ecology when those resources are more similar. Clustered communication resources means that individuals are more likely to use these different communication resources together, as the use of one communication resource in the cluster may be associated with (or lead to, and or result from) the use of another. Similarly, when communication resources are less clustered with other resources, they are less likely to be used with other resources. As discussed previously, our results indicate the most central communication resources (partisan communication resources and local organizations) in the ecology were not the communication resources that were most frequently used to find out about COVID-19 (television news). In this way, a network analysis approach to analyzing a communication ecology illustrates something about the nature of communication resource use that is not available from other more traditional metrics (e.g., descriptive statistics, factor analysis). As such, it provides another methodological tool in the toolbox.

It is also worth emphasizing that a CEN structure is the product of the communication resources that are included (or omitted) in the analysis. Additionally, communication ecologies may vary based on the purpose (or goal) of the ecology. Thus, the communication ecology identified here might not be the same as a communication ecology related to another public health topic (e.g., the ongoing opioid crisis in the United States) Moreover, COVID-19 communication ecologies are likely to change over the course of the pandemic. Our results are based on data collected in September 2020, and analyses of communication ecologies earlier or later in the crisis might indicate differences in the COVID-19 communication ecology overall.

### Limitations

Like all research, this project was subject to several limitations. First, our data were collected at one time and thus represent a cross-sectional view of communication behaviors in two U.S. states. To better understand the interactions and interdependencies in a communication ecology, longitudinal data are needed to examine how relationships between communication behaviors strengthen or weaken over time. Second, our participants represent a convenience sample and thus our results cannot be generalized back to the population. Third, our exploration of the COVID-19 communication ecology was limited to the items we assessed, and thus other possible communication resources are not represented. Fourth, a comparison of communication ecologies (network) was not included in our analysis and should be included in future research. In the case COVID-19, it would be useful to compare the communication ecologies of liberals and conservatives or younger and older individuals as there may be meaningful differences.

## Conclusion

The COVID-19 outbreak began in December 2019 and soon became a global pandemic, resulting in major public health consequences for countries across the world. During this time, individuals have been challenged to understand the risk of COVID-19 and to identify ways to stay safe. Much of this learning occurred through COVID-19 communication ecologies that consist of interpersonal, organizational, and mediated communication resources. In this study we examined the COVID-19 communication ecology in two U.S. states in September 2021. We introduced the CEN model, which posits that similarly useful communication resources will cluster in a communication ecology, and used network analysis for visualization of the ecology. Our results indicate a robust COVID-19 communication ecology, in which partisan and organizational communication resources were identified as being most important. We also identified five clusters within the COVID-19 communication ecology, and explored effects of cluster usage on beliefs in COVID-19 misinformation. Our use of network analysis illustrates benefits of this analytical approach to studying communication ecologies.
